# Physician advice on avoiding secondhand smoke exposure and referrals for smoking cessation services

**DOI:** 10.1186/1617-9625-10-10

**Published:** 2012-07-02

**Authors:** Judy Kruger, Angela Trosclair, Abby Rosenthal, Steve Babb, Robert Rodes

**Affiliations:** 1 Centers for Disease Control and Prevention, Office on Smoking and Health, 4770 Buford Highway, M/S-K-50, Atlanta, GA, 30341, USA

**Keywords:** Secondhand smoke, Cessation, Physician, Advice and Referral

## Abstract

**Background:**

Secondhand smoke (SHS) exposure causes premature death and disease. Eliminating smoking in indoor spaces is the only way to fully protect nonsmokers from SHS exposure, and also contributes to helping smokers quit smoking. Primary health care providers can play an important role in advising nonsmoking patients to avoid SHS exposure, cautioning current smokers against exposing others to SHS, and referring tobacco users to cessation programs.

**Methods:**

The purpose of this paper is to examine primary care provider (obstetricians/gynecologists, pediatricians, and general practitioners) advice regarding SHS exposure and referral to cessation programs. Using data from the 2008 DocStyles survey (n = 1,454), we calculated the prevalence and adjusted odds ratios for offering patients advice regarding SHS exposure and referring adults who smoked or used other tobacco products to a cessation program.

**Results:**

The current study found that among a convenience sample of primary care providers, 94.9% encouraged parents to take steps to protect children from SHS exposure, 86.1% encouraged smokers to make their homes and cars smoke-free, and 77.4% encouraged nonsmokers to avoid SHS exposure. Approximately 44.0% of primary care providers usually or always referred patients who smoked or used tobacco products to cessation programs such as a quitline, a group cessation class, or one-on-one counseling.

**Conclusion:**

Findings from a convenience sample of primary care providers who participated in a web-based survey, suggests that many primary care providers are advising parents to protect children from SHS exposure, encouraging patients who smoke to maintain smoke-free homes and cars, and advising smokers on ways to avoid exposing others to SHS. Healthcare providers are encouraged to advise patients to avoid SHS exposure and to refer patients who use tobacco products to cessation services.

## Background

There is strong evidence that exposure to secondhand smoke (SHS) is harmful to people. It causes heart disease and lung cancer in nonsmoking adults [1-3], and sudden infant death syndrome, acute respiratory infections, ear infections, worsened asthma symptoms, and other health conditions in children [1,3]. Research suggests that 100% smoke-free indoor air environments are the only effective way to fully protect nonsmokers from SHS exposure [1,4]. Additionally, smoke-free environments and policies have been found to encourage current smokers to quit [1,3].

In response to growing concerns about the health effects of SHS, as discussed in the 2006 Surgeon General’s Report on the health consequences of involuntary exposure to tobacco smoke [1], and the number of studies reporting that smoke-free laws were associated with rapid and substantial reductions in heart attack hospitalizations, the Institute of Medicine (IOM) conducted a review on the plausibility of these findings. Upon completion of the review, the IOM published a report which concluded that even brief exposure to SHS could trigger a heart attack and that smoke-free laws reduce their occurrence [5]. These investigations helped to shed light on the need for more patient counseling about SHS exposure and the deleterious health effects of passive smoking.

Clearly, treating tobacco use and dependence should be a high priority for physicians as well as for all those who organize, provide, and pay for healthcare [6]. Prior studies have used currently available healthcare criteria that included patient advice to quit and a discussion of smoking cessation medications and cessation strategies during the office visit. In the Medicaid population, the proportion of smokers who received advice to quit from a physician increased from 65.6% in 2005 to 69.3% in 2008 [7]. State Medicaid cessation coverage is gradually expanding, with 47 states offering coverage for tobacco-dependence treatment as of 2009 [8].

Offering help to quit tobacco use is 1 of 6 evidence-based tobacco-control strategies included in the World Health Organization’s MPOWER package [2]. Specifically, healthcare providers are urged to incorporate cessation advice into primary care settings and practice [2]. In the US Public Health Service guideline *Treating Tobacco Use and Dependence: 2008 Update*, Fiore and colleagues concluded that, in order for primary care providers to intervene with tobacco users, there needs to be ample institutional support by clinicians, administrators, insurers, and purchasers [9]. The Task Force on Community Preventive Services [10] has published updated guidelines on tobacco prevention to assist healthcare providers in incorporating counseling on cessation and reduction of exposure to SHS into standard care. Despite the existence of national guidelines [9-11], limited information is available on the extent to which healthcare providers are promoting such services.

Advice from healthcare providers to their patients to avoid SHS exposure and to quit smoking can broaden population-based support for smoke-free environments and reduce smoking rates. This study examines physician advice regarding avoidance of SHS exposure and referral to a smoking cessation program.

## Methods

### Study design

We selected our study population from respondents to the 2008 DocStyles survey, which was conducted by Porter Novelli, a social marketing and public relations firm. DocStyles is an annual web survey that provides insight into physicians’ attitudes, behaviors, knowledge, and counseling behaviors on health issues, and assesses their use and trust of available health information sources. The sampling was conducted by Epocrates, Inc. using respondents identified from the Epocrates Honors Panel, an opt-in, verified panel of 135,000 medical practitioners. The primary recruitment method was based on healthcare providers’ self-selection to join the panel and complete the online healthcare survey at http://www.epocrates.com/honors, after receiving an initial email from Epocrates.

Eligible physician verification was achieved by checking each physician’s first name, last name, date of birth, medical school, and graduation date against the American Medical Association’s (AMA) master file at the time of panel registration. Physicians were screened to include only those who practice in the US; actively see patients; work in an individual, group, or hospital practice; and have been practicing medicine for at least 3 years. Epocrates randomly selected a sample of eligible physicians from their main database to load into their invitation database. In order for Epocrates to reach the needed pre-determined sample size for the current study, 14,346 physicians were invited to participate. Of those invited to participate in the DocStyles survey, 1,880 completed the entire survey, 33 did not complete the entire survey, 141 did not meet the screening criteria, 1,088 logged in to take the survey but were terminated due to filled quotas for their specialty, and 11,204 did not respond to the invitation or tried to respond after the survey closed, resulting in a response rate of 22%. The response rate http://www.researchinfo.com/docs/calculators/response.cfm was calculated by weighting respondents who were terminated due to filled quotas as a factor of the overall sample pool [12]. The sample was drawn to match AMA master file proportions for age, gender, and region. In 2008, the goal was to recruit 1,000 primary care physician (family physicians, general practitioners, internists), 250 pediatricians, 250 obstetricians/gynecologists (OB/GYN), 250 dermatologists and 130 registered dieticians. The different physician specialties were included because there were of particular interest to the data collectors and the total sample by itself was not intended to be representative of the national population of physicians or physician specialties. Physicians were paid an honorarium of $50-$75 for completing the survey. Respondents were not required to participate in the 140-question survey, which had multiple subparts designed to provide insights into physicians’ counseling behaviors, and were able to exit the survey at any point.

### Study variables

Primary healthcare provider personal characteristics consisted of sex, age (18–35, 36–45, 46–55, and ≥56), race/ethnicity (white, black, Hispanic, Asian, other), and smoking status. Smoking status was dichotomized into current smokers (smoked 1 to 7 days/week) and nonsmokers since lifetime use of cigarettes was not obtained. Professional characteristics included years in practice, number of doctors in practice, type of practice (individual practice, group practice, hospital/clinic practice), number of patients seen per week, and whether they maintained teaching privileges.

The 2008 DocStyles survey included a series of questions on provider practices regarding giving health-related advice. Respondents were first asked whether they advised parents or guardians to keep their children from being exposed to smoke from cigarettes or other tobacco products. If they answered yes, they were asked (1) whether they advised patients who do not smoke tobacco or use other tobacco products to avoid being exposed to SHS, and (2) whether they advised their patients who smoke tobacco or use other tobacco products to create smoke-free homes and cars (i.e., not to smoke or allow smoking inside their homes or cars at any time). A single question was used to determine whether healthcare providers referred patients who smoked or used tobacco products to cessation programs such as a telephone quitline, a smoking cessation class, or one-on-one counseling. Respondents were asked to select one response from the possible options (always, usually, sometimes, rarely, never). In the logistic regression analyses, these responses were dichotomized into “always/usually” vs. “sometimes/never/rarely.”

### Statistical analyses

Advice and referral practices were examined using the entire sample from the 2008 DocStyles survey. The primary outcomes of interest were advice regarding avoidance of SHS exposure, and referral to a smoking cessation program. Because there was no significant difference between the entire sample (consisting of OB/GYNs, pediatricians, internists, general practitioners, dermatologists, and registered dieticians) and the selected sample of primary care providers (internists, general practitioners, pediatricians and OB/GYNs) on the outcomes of interest, this paper focused on the selected sample. We excluded respondents who were not family practitioners, general practitioners, internists, obstetricians/gynecologists or pediatricians, and those who were missing data on demographic characteristics or did not respond to all of the questions of interest. The final analytic sample consisted of 1,454 primary care providers. Of the 1,454 physicians included in the current analysis, 496 were family/general practitioners, 473 were internists, 244 were pediatricians, and 241 were OB/GYNs.

Descriptive statistics of personal and practice characteristics were used to characterize primary care providers in the selected sample population. The analysis focused on calculating the prevalence and odds of primary care providers’ providing advice on avoiding SHS exposure and referring smokers to smoking cessation resources. A 2-sided *t*-test with an alpha level of *P* < 0.05 was used to determine the statistical significance. Logistic regression was used to adjust for healthcare provider characteristics (sex, age, and race/ethnicity). Separate models were analyzed initially for patient smoking status (smoker vs. nonsmoker) as a confounder or effect modifier on SHS avoidance advice and cessation referral. All analyses were performed using SAS version 9.2 (SAS Institute Inc., Cary, North Carolina).

## Results

Frequencies for primary care provider personal and professional characteristics are provided in Table 1. Respondents were more commonly men (75.1%), aged 36–45 years (40.6%), and nonsmokers (93.3%). One-third of the sample self-identified as family/general practitioners (34.1%). Over half the sample maintained teaching privileges (57.0%). Approximately two-thirds of the sample physicians had been in practice 6–20 years (64.2%), one-third worked in practices with 3–5 physicians (27.2%), and one-third consulted with 76–100 patients per week (34.4%). The final weighted sample was comparable to the AMA master file, in terms of gender (74.0% men), average age (45 years), and years in practice (13.1 years) (data not shown).

**Table 1:** Personal and practice characteristics of primary care providers— DocStyles Survey, 2008

**Physician Characteristics**	**Total**	**Men**	**Women**
	**(n = 1,454)**	**(n = 1,092)**	**(n = 362)**
	**(%)**	**(%)**	**(%)**
**Age**			
26-35	17.9	15.7	24.6
36-45	40.6	39.7	43.1
46-55	28.3	29.4	25.1
≥56	13.2	15.2	7.2
**Race/ethnicity**			
White	72.0	73.4	67.7
Black	3.4	2.6	5.8
Hispanic	4.3	4.3	4.4
Asian	15.7	15.0	18.0
Other	4.5	4.7	4.1
**Smoking status**			
Smoker	6.7	8.3	1.7
Nonsmoker	93.3	91.7	98.3
**Health care provider**			
Family/general practitioners	34.1	34.3	33.7
Internists	32.5	34.4	26.5
Pediatricians	16.8	14.1	24.9
Obstetricians/gynecologists	16.6	17.2	14.9
**Teaching privileges**			
Yes	57.0	57.4	55.8
No	43.0	42.6	44.2
**Type of practice**			
Individual	16.7	17.9	13.0
Group	64.0	64.7	61.9
Hospital/clinic	19.3	17.3	25.1
**Years in practice**			
0-5	13.5	11.9	18.5
6-10	31.8	30.7	35.1
11-20	32.4	31.6	34.8
≥21	22.3	25.8	11.6
**Number of physicians in practice**			
1-2	26.0	26.5	24.6
3-5	27.2	27.2	27.1
6-10	22.5	21.9	24.6
11-25	12.9	13.0	12.4
≥26	11.5	11.5	11.3
**Number of patients per week**			
1-75	21.3	17.9	31.2
76-100	34.4	32.5	40.1
101-150	32.8	36.4	22.1
≥151	11.6	13.2	6.6
Total	—	75.1	24.9

Table 2 describes advice provided by primary care providers on avoiding SHS exposure. Almost ninety-five percent (94.9%) of primary care providers reported encouraging parents to take steps to protect children from SHS exposure, 86.1% reported encouraging smokers to maintain smoke-free homes and cars, and 77.4% reported encouraging nonsmokers to avoid SHS exposure. Advice on SHS was most common among primary care providers who were women, those ≤5 years in practice, and those who see ≥151 patients per week.

**Table 2:** Types of primary care providers’ advice about exposure to secondhand smoke—DocStyles Survey, 2008

**Physician**	**Encouraged protecting children from SHS exposure**		**Encouraged smokers to maintain smoke-free homes/cars**		**Encouraged nonsmokers to avoid SHS exposure**	
**Characteristics**	**N**	**% (95%C.I.)**	**N**	**% (95%C.I.)**	**N**	**% (95%C.I.)**
**Sex**						
Men	1,028	94.1 (91.8-96.4)	925	84.7 (82.3-87.1)	828	75.8 (73.2-78.4)
Women	352	97.2 (94.9-99.5)	327	90.3 (87.9-92.8)	297	82.0 (79.5-84.6)
**Age**						
26-35	250	96.2 (94.1-98.2)	230	88.5 (86.3-90.6)	199	76.5 (74.3-78.8)
36-45	562	95.3 (92.7-97.8)	513	86.9 (84.2-89.7)	466	79.0 (76.1-81.9)
46-55	385	93.4 (91.1-95.8)	353	85.7 (83.2-88.2)	311	75.5 (72.9-78.1)
≥56	183	95.3 (93.5-97.1)	156	81.3 (79.4-83.1)	149	77.6 (75.6-79.6)
**Race/ethnicity**						
White	987	94.3 (91.9-96.7)	901	86.1 (83.6-88.5)	787	75.2 (72.5-77.8)
Black	47	95.9 (95.0-96.9)	42	85.7 (84.7-86.7)	36	73.5 (72.4-74.5)
Hispanic	61	96.8 (95.7-97.9)	53	84.1 (83.0-85.2)	56	88.9 (87.6-90.2)
Asian	221	96.5 (94.6-98.4)	198	86.5 (84.4-88.5)	187	81.7 (79.5-83.8)
Other	64	97.0 (95.9-98.1)	58	87.9 (86.7-89.0)	59	89.4 (88.1-90.7)
**Smoking status**						
Smoker	96	99.0 (97.6-100))	82	84.5 (83.2-85.9)	74	76.3 (74.8-77.7)
Nonsmoker	1,284	94.6 (93.3-96.0)	1,170	86.2 (84.8-87.6)	1,051	77.5 (76.0-78.9)
**Health care provider**						
Family/general practitioners	485	97.8 (96.5-99.1)	439	88.5 (85.7-91.3)	382	77.0 (73.3-80.7)
Internists	442	93.6 (91.4-95.8)	395	83.7 (80.4-87.0)	379	80.3 (76.7-83.9)
Pediatricians	242	99.2 (98.0-100)	236	96.7 (94.5-99.0)	211	86.5 (82.2-90.8)
Obstetricians/gynecologists	211	87.2 (83.0-91.4)	182	75.2 (69.8-80.6)	153	63.2 (57.1-69.3)
**Teaching privileges**						
Yes	783	94.5 (91.8-97.1)	714	86.1(83.4-88.9)	648	78.2 (75.3-81.1)
No	597	95.5 (92.9-98.1)	538	86.1(83.3-88.8)	477	76.3 (73.4-79.2)
**Type of practice**						
Individual	233	95.9 (93.9-97.9)	204	84.0 (81.9-86.0)	196	80.7 (78.4-82.9)
Group	880	94.5 (92.0-97.1)	806	86.6 (83.9-89.2)	705	75.7 (72.9-78.6)
Hospital/clinic	267	95.4 (93.3-97.4)	242	86.4 (84.2-88.6)	224	80.0 (77.7-82.3)
**Years in practice**						
0-5	192	97.5 (95.6-99.3)	176	89.3 (87.4-91.3)	156	79.2 (77.2-81.2)
6-10	445	96.3 (93.9-98.8)	410	88.7 (86.1-91.3)	361	78.1 (75.4-80.9)
11-20	436	92.6 (90.1-95.0)	397	84.3 (81.7-86.9)	357	75.8 (73.1-78.5)
≥21	307	94.8 (92.6-96.9)	269	83.0 (80.7-85.3)	251	77.5 (75.0-79.9)
**Number of physicians in practice**						
1-2	357	94.4 (92.1-96.8)	315	83.3 (80.9-85.7)	294	77.8 (75.2-80.3)
3-5	373	94.4 (92.1-96.8)	343	86.8 (84.4-89.3)	306	77.5 (74.9-80.1)
6-10	313	95.7 (93.5-97.9)	291	89.0 (86.7-91.3)	256	78.3 (75.8-80.7)
11-25	178	95.2 (93.4-97.0)	160	85.6 (83.7-87.4)	145	77.5 (75.6-79.5)
≥26	159	95.2 (93.5-96.9)	143	85.6 (83.9-87.4)	124	74.3 (72.4-76.1)
**Number of patients per week**						
1-75	296	95.8 (93.6-98.0)	265	85.8 (83.5-88.0)	238	77.0 (74.6-79.4)
76-100	464	92.8 (90.3-95.3)	424	84.8 (82.2-87.4)	369	73.8 (71.1-76.5)
101-150	457	95.8 (93.3-98.3)	413	86.6 (84.0-89.2)	381	79.9 (77.1-82.6)
≥151	163	97.0 (95.3-98.7)	150	89.3 (87.5-91.1)	137	81.5 (79.6-83.5)
Total	1,380	94.9	1,252	86.1	1,125	77.4

SHS = secondhand smoke.

C.I. = confidence interval.

Logistic regression analysis showed that female primary care providers were more likely than their male peers to counsel patients about avoiding SHS exposure (Table 3). Primary care providers who were Hispanic (AOR: 2.61; 95% CI: 1.18-5.80), Asian (AOR: 1.47; 95% CI: 1.02-2.13) or from another racial/ethnic group (AOR: 2.87; 95% CI: 1.28-6.41) were more likely to encourage nonsmokers to avoid exposure to SHS than whites. Internists (AOR: 0.31; 95% CI: 0.15-0.62) were less likely than family/general practitioners to encourage parents to take steps to protect children from SHS exposure. Primary care providers who were pediatricians were more likely to encourage smokers to maintain smoke-free homes and cars (AOR: 3.69; 95% CI: 1.72-7.92) and to encourage nonsmokers to avoid SHS exposure (AOR: 1.82; 95% CI: 1.08-2.79) than family/general practitioners. Obstetricians/gynecologists were less likely than family/general practitioners to counsel patients about avoiding SHS exposure.

**Table 3:** Primary care providers’ advice about exposure to secondhand smoke—DocStyles Survey, 2008

**Physician**	**Encouraged protecting children from SHS exposure**		**Encouraged smokers to maintain smoke-free homes/cars**		**Encouraged nonsmokers to avoid SHS exposure**	
**Characteristics**	**AOR**	**95%C.I.**	**AOR**	**95%C.I.**	**AOR**	**95%C.I.**
**Sex**						
Men	1.0	—	1.0	—	1.0	—
Women	2.14	1.10-4.17	1.62	1.10-2.39	1.49	1.09-2.03
**Age**						
26-35	1.0	—	1.0	—	1.0	—
36-45	0.88	0.42-1.84	0.90	0.58-1.42	1.24	0.87-1.77
46-55	0.68	0.33-1.40	0.82	0.51-1.31	1.09	0.75-1.57
≥56	1.03	0.42-2.54	0.61	0.36-1.04	1.29	0.82-2.03
**Race/ethnicity**						
White	1.0	—	1.0	—	1.0	—
Black	1.23	0.29-5.24	0.86	0.38-1.95	0.85	0.44-1.63
Hispanic	1.81	0.43-7.56	0.82	0.41-1.67	2.61	1.18-5.80
Asian	1.56	0.74-3.26	0.95	0.62-1.45	1.47	1.02-2.13
Other	1.89	0.46-7.85	1.11	0.52-2.38	2.87	1.28-6.41
**Smoking status**						
Smoker	3.98	0.81-4.18	0.94	0.53-1.69	0.96	0.59-1.56
Nonsmoker	1.0	—	1.0	—	1.0	—
**Health care provider**						
Family/general practitioners	1.0	—	1.0	—	1.0	—
Internists	0.31	0.15-0.62	0.68	0.46-1.00	1.17	0.85-1.61
Pediatricians	2.40	0.52-11.02	3.69	1.72-7.92	1.82	1.18-2.79
Obstetricians/gynecologists	0.15	0.08-0.31	0.40	0.27-0.60	0.52	0.37-0.73
**Teaching privileges**						
Yes	1.0	—	1.0	—	1.0	—
No	1.27	0.78-2.08	0.99	0.73-1.35	0.90	0.70-1.16
**Type of practice**						
Individual	1.38	0.60-3.20	0.95	0.58-1.56	1.09	0.70-1.71
Group	0.99	0.54-1.84	1.09	0.73-1.63	0.84	0.59-1.17
Hospital/clinic	1.0	—	1.0	—	1.0	—
**Years in practice**						
0-5	2.61	0.73-9.28	1.68	0.75-3.78	0.97	0.50-1.86
6-10	1.61	0.63-4.12	1.55	0.80-3.00	0.84	0.50-1.42
11-20	0.70	0.34-1.42	0.97	0.60-1.58	0.78	0.51-1.19
≥21	1.0	—	1.0	—	1.0	—
**Number of physicians in practice**						
1-2	1.0	—	1.0	—	1.0	—
3-5	0.96	0.52-1.79	1.29	0.86-1.93	0.97	0.68-1.36
6-10	1.26	0.63-2.54	1.54	0.99-2.40	1.02	0.71-1.46
11-25	1.06	0.47-2.38	1.13	0.69-1.86	0.93	0.61-1.43
≥26	1.13	0.49-2.63	1.13	0.67-1.91	0.81	0.52-1.24
**Number of patients per week**						
1-75	0.58	0.20-1.67	0.63	0.35-1.14	0.69	0.43-1.12
76-100	0.35	0.14-0.92	0.61	0.35-1.06	0.60	0.38-0.93
101-150	0.70	0.26-1.89	0.77	0.44-1.34	0.89	0.57-1.39
≥151	1.0	—	1.0	—	1.0	—

SHS = secondhand smoke.

AOR = odds ratio adjusted for sex, age, and race/ethnicity; odds ratios compare yes to no answer for each item.

C.I. = confidence interval.

Table 4 shows the prevalence of primary care referral of a smoker or tobacco user to a tobacco cessation program. Referral by primary care providers was most common among providers 36–45 years of age (46.1%), those who were classified as other race/ethnicity (51.5%), those who were family/general practitioners (50.8%), those with teaching privileges (45.5%), those who worked in a hospital or clinic practice (50.4%), and those who were in a practice with ≥11 physicians (49.7%). Primary care providers who were classified as other racial/ethnic groups (AOR: 1.41; 95% CI: 1.85-2.34) were more likely to usually/always refer tobacco users to a cessation program than whites. Internists (AOR: 0.74; 95% CI: 0.57-0.96) and pediatricians (AOR: 0.39; 95% CI: 0.28-0.54) were less likely to refer patients to cessation programs than were family/general practitioners. Those who worked in group practices (AOR: 0.75; 95% CI: 0.57-1.00) were less likely to refer patients to cessation programs than primary care providers who work in hospitals or clinics.

**Table 4:** Prevalence and odds of referral of primary care providers to a cessation program—DocStyles Survey, 2008

**Physician**	**Referral provided: Usually/Always**			**Referral provided: Sometimes/Never/Rarely**			**Odds of referral**	
**Characteristics**	**n**	**%**	**95%C.I.**	**n**	**%**	**95%C.I.**	**AOR**	**95%C.I.**
**Sex**								
Men	467	42.8	39.9-45.7	625	57.2	54.3-60.1	1.0	—
Women	172	47.5	42.4-52.7	190	52.5	47.3-57.6	1.19	0.93-1.52
**Age**								
26-35	117	43.8	37.9-49.9	146	56.2	50.1-62.1	1.0	—
36-45	272	46.1	42.1-50.1	318	53.9	49.9-57.9	1.13	0.84-1.52
46-55	185	44.9	40.2-49.7	227	55.1	50.3-59.8	1.10	0.80-1.52
≥56	68	35.4	29.0-42.4	124	64.6	57.6-71.0	0.76	0.51-1.12
**Race/ethnicity**								
White	447	42.7	39.7-45.7	600	57.3	54.3-60.3	1.0	—
Black	20	40.8	28.1-55.0	29	59.2	45.0-71.9	0.88	0.49-1.58
Hispanic	31	49.2	37.1-61.4	32	50.8	38.6-62.9	1.27	0.76-2.11
Asian	107	46.7	40.3-53.2	122	53.3	46.8-59.7	1.15	0.85-1.54
Other	34	51.5	39.6-63.3	32	48.5	36.7-60.4	1.41	1.85-2.34
**Smoking status**								
Smoker	46	47.7	37.7-57.3	51	52.6	42.7-62.3	1.18	0.78-1.79
Nonsmoker	593	43.7	41.1-46.4	764	56.3	53.6-58.9	1.0	—
**Health care provider**								
Family/general practitioners	252	50.8	46.4-55.2	244	49.2	44.8-53.6	1.0	—
Internists	207	43.9	39.4-48.4	265	56.1	51.6-60.6	0.74	0.57-0.96*
Pediatricians	73	29.9	24.5-36.0	171	70.1	64.0-75.5	0.39	0.28-0.54**
Obstetricians/gynecologists	107	44.2	38.1-50.5	135	55.8	49.5-61.9	0.79	0.58-1.08
**Teaching privileges**								
Yes	377	45.5	42.1-48.9	452	54.5	51.1-57.9	1.0	—
No	262	41.9	38.1-45.8	363	58.1	54.2-61.9	0.86	0.70-1.06
**Type of practice**								
Individual	99	40.7	34.7-47.0	144	59.3	53.0-65.3	0.70	0.49-0.1.01
Group	399	42.9	39.7-46.1	532	57.1	53.9-60.3	0.75	0.57-1.00*
Hospital/clinic	141	50.4	44.5-56.2	139	49.6	43.8-55.5	1.0	—
**Years in practice**								
0-5	92	46.7	39.8-53.7	105	53.3	46.3-60.2	1.45	0.84-2.50
6-10	214	46.3	41.8-50.9	248	53.7	49.1-58.2	1.33	0.84-2.10
11-20	209	44.4	39.9-48.9	262	55.6	51.1-60.1	1.14	0.78-1.66
≥21	124	38.3	33.1-43.7	200	61.7	56.3-60.1	1.0	—
**Number of physicians in practice**								
1-2	152	40.2	35.4-45.2	226	59.8	54.8-64.6	1.0	—
3-5	165	41.8.	37.0-46.7	230	58.2	53.3-63.0	1.06	0.79-1.41
6-10	146	44.6	39.3-50.1	181	55.4	49.9-60.7	1.18	0.87-1.60
11-25	93	49.7	42.6-56.9	94	50.3	43.1-57.4	1.43	1.00-2.05*
≥26	83	49.7	42.2-57.2	84	50.3	42.8-57.8	1.43	0.99-2.08
**Number of patients per week**								
1-75	138	44.7	39.2-50.3	171	55.3	49.7-60.8	0.97	0.66-1.43
76-100	215	43.0	38.7-47.4	285	57.0	52.6-61.3	0.90	0.63-1.29
101-150	211	44.2	39.8-48.7	266	55.8	51.3-60.2	0.97	0.68-1.39
≥151	75	44.6	37.3-52.2	93	55.4	47.8-62.7	1.0	—
Total	639	43.9	41.4-46.5	815	56.1	53.5-58.6	—	—

AOR = odds ratio adjusted for sex, age, and race/ethnicity; odds ratios compare yes to no answer for each item.

C.I. = confidence interval.

*P < 0.05.

**P < 0.001.

## Discussion

The current study provides novel findings on advice given by a convenience sample of primary care providers regarding avoidance of SHS exposure and referral to a smoking cessation program. In this sample, a large proportion of primary care providers reported encouraging their patients to protect children from SHS, to make their homes and cars smoke-free, and to avoid SHS exposure. Although there may be differences between this opt-in web-based physician sample and the full universe of primary care providers, these findings suggest that many health care providers provide SHS counseling in clinical practice. Since SHS causes premature death and disease in both children (especially asthmatics) [1] and adults (especially those with respiratory conditions, those at increased risk for heart disease, or those with a history of heart disease) [1,5], health care providers are encouraged to counsel smokers and nonsmokers on the risks of SHS exposure. In essence, the US Public Health Service’s updated publication [9] is a clinical practice guide for all clinicians, and it serves as the basis for specific sub-specialty groups that may prepare specific guides for their organizations. Although this guide does not universally address SHS exposure, it emphasizes the importance of the clinician’s role in managing tobacco use by encouraging patients to avoid SHS as part of the medical visit. The American Academy of Pediatrics encourages clinicians to be active in eliminating tobacco use and SHS exposure of children [13], and although the US Preventive Services Task Force does not have a specific recommendation to prevent SHS exposure, they do have recommendations for smoking cessation among adults and young people [14].

Exposure to SHS has been found to be harmful to adults and children alike, and nonsmokers are frequently exposed to SHS in homes, workplaces, vehicles, and public places [15,16]. Healthcare providers are in a unique role to raise awareness of the negative health effects of SHS, and may consider incorporating elements from the framework and intervention known as CEASE, the Clinical Effort Against Secondhand Smoke Exposure [17]. Williams and colleagues (2005) suggest that clinicians should actively engage in screening and SHS counseling with all of their patients who use tobacco and that intervening with nonsmokers to create smoke-free homes may help increase cessation among smokers [18]. In our study, 80.0% of primary care providers in hospitals and clinics encouraged nonsmokers to avoid SHS exposure, and 86.4% encouraged smokers to maintain smoke-free homes and cars. However, in communities and states that have yet to enact comprehensive smoke-free laws [19], patient education by healthcare providers on the dangers posed by SHS and on the importance of avoiding locations where smoking is allowed could contribute to the expansion of smoke-free environments by changing people’s expectations and behavior regarding smoking in public places, and motivating parents to protect their children [20].

This analysis found that, among this convenience sample, only 43.9% of primary care providers referred patients who smoked or used tobacco products to a cessation program, a figure somewhat higher than the 25.9% reported in a 2006 US study [21]. It is likely that our findings may be attributable in part to a ‘healthy respondent’ effect, since 93.3% of physicians who responded to the survey were nonsmokers, and might therefore be more likely to advise patients to avoid SHS exposure and to quit smoking. However, research shows that the proportion of health professionals who smoke has decreased over time [22]. Referral to cessation services is only one part of the clinical practice guidelines for smoking cessation [9], and providing tobacco cessation advice for those who use tobacco products may not be appropriate for all patients. In addition, there is some uncertainty as to the validity of this measure as an isolated indicator for quality of service for patients who smoke. Some physicians may elect to provide cessation treatment themselves rather than refer patients for counseling, and some smokers not interested in quitting may not be appropriate for referral to resources that focus on supporting cessation attempts.

Practice size is likely to influence referral patterns as well. In our study, smaller practices (individual or group) were less likely to refer patients to cessation services than larger practices (hospital or clinic); in larger clinical settings, there are perhaps more resources available to facilitate adherence to smoking cessation guidelines, and there may be greater potential for coordination of services. Regardless of practice size, referral to smoking cessation services may require addressing the obstacles identified by healthcare providers, including lack of time (especially for counseling), lack of availability or awareness of resources, lack of adequate reimbursement, and competing demands for other services.

There are several limitations to this study. First, the sampling methodology that was used for the survey drew from a self-selected group (Epocrates Honor Panel) and, thus, resulted in a convenience sample. Although the method used quotas and weighting to produce a dataset that matched the specialty breakdown of the AMA membership, the findings may not be representative of all primary care providers in the US, especially those who were not members of the AMA. Thus, findings may not reflect the primary health care reality in terms of being nationally representative. Second, response rates for 2008 were lower than for previous years, which may also affect the representativeness of participants. This may be because the survey was almost twice as long as in previous years and potential participants were informed of the survey length in the invitational email, which may have dissuaded participation. Third, the questions did not address the type or amount of tobacco products patients were using, and the questions used to ascertain provider advice on avoiding SHS exposure and referral to cessation services had not been formally validated. The questions relied on recall over the previous 12 months and it is possible that providers failed to remember providing advice or referral. Thus, questions may not accurately capture providers’ actual behavior. Fourth, brief counseling is multifaceted, and there are distinctions between asking, advising, providing assistance, and referring in clinical practice. Referral may not be appropriate for all patients, since this is only one way to fulfill components of the “5 As” (ask, advise, assess, assist, arrange follow-up) of the clinical practice guidelines for smoking cessation [9]. In addition, providers using self-reporting tend to over-report behaviors that they assume they should be doing [18]. However, this tendency toward high self-reported response rates has been found in other studies such as those examining counseling and referral to outpatient psychiatry and clinical psychology [23]. Fifth, in this survey, respondents were asked only about advising patients to avoid SHS exposure and referring them to cessation services. An expanded list of survey responses for specific evidence-based cessation services may have identified specific service preferences (e.g., quitline, group cessation class, one-on-one counseling, clinic check-backs). Future research should utilize other measures of physician behavior such as post-visit patient surveys, chart audits, and direct observation.

## Conclusions

The findings of this web-based survey provide a glimpse into primary care providers’ practices regarding advising nonsmokers to avoid SHS exposure and referring smokers to a smoking cessation program. We observed that many providers in this sample are advising their patients to take steps to protect themselves and their children from SHS exposure. They also appear to be identifying patients who use tobacco products and who want to quit and referring them to cessation resources. These resources could potentially include in-clinic follow-up, the toll-free phone number 1-800-QUIT-NOW, which transfers callers to their state quitlines, or the National Cancer Institute cessation website http://www.smokefree.gov.

Consistent education and advice on SHS from providers would increase patients’ awareness of the serious health effects of SHS and motivate them to avoid SHS exposure. In addition to prompting individual behavior change, SHS counseling could play an important role in spurring broader population-level efforts to expand smoke-free environments and in changing public attitudes regarding the social acceptability of smoking. These combined individual-level and population-level effects could yield significant reductions in child and adult morbidity and mortality, especially among high-risk groups such as children with asthma and adults with heart disease or respiratory conditions.

## Abbreviations

SHS, Refers to secondhand smoke; IOM, Refers to Institute of Medicine; AMA, Refers to the American Medical Association; OB/GYN, Refers to obstetricians/gynecologists; CI, Refers to confidence interval; AOR, Refers to adjusted odds ratio..

## Competing interests

None of the authors have competing interests. The findings and conclusions in this report are those of the authors and do not necessarily represent the official position of the Centers for Disease Control and Prevention.

## Authors’ contributions

JK conceived the research idea and held the primary responsibility for drafting the manuscript. AT participated in the analysis and interpretation of the data. AR, SB and RR participated in finalizing the study design, reviewing the literature and writing the manuscript. All authors read and approved the final manuscript.
